# Factors Associated with Stunting among Children Aged 0 to 59 Months from the Central Region of Mozambique

**DOI:** 10.3390/nu9050491

**Published:** 2017-05-12

**Authors:** Loida María García Cruz, Gloria González Azpeitia, Desiderio Reyes Súarez, Alfredo Santana Rodríguez, Juan Francisco Loro Ferrer, Lluis Serra-Majem

**Affiliations:** 1 Research Institute of Biomedical and Health Sciences, University of Las Palmas de Gran Canaria, 35016 Las Palmas de Gran Canaria, Spain; loidagc@gmail.com (L.M.G.C.); pediatria@dcc.ulpgc.es (G.G.A.); dreysua@gmail.com (D.R.S.); 2 Clinical Genetics Unit, Complejo Hospitalario Insular-Materno Infantil, 35016 Las Palmas de Gran Canaria, Spain; consultageneticasantana@gmail.com; 3 Department of Clinical Sciences, University of Las Palmas de Gran Canaria, 35016 Las Palmas de Gran Canaria, Spain; juanfrancisco.loro@ulpgc.es; 4 Ciber OBN (CB06/03), Instituto Carlos III, Spanish Government, 28029 Madrid, Spain; 5 Research Institute of Biomedical and Health Sciences, University of Las Palmas de Gran Canaria/Nutrition Without Borders, 35016 Las Palmas de Gran Canaria, Spain

**Keywords:** undernutrition, growth retardation, stunting, nutritional assessment, mozambique

## Abstract

The objective of this study was to identify the major socio-demographic, health, and environmental determinants of stunting among children aged 0–59 months from the Tete province (Mozambique) and offering useful information for future healthcare strategies and interventions. A case-control study was conducted among 282 (162 boys; 120 girls) children under five years of age from the central region of Mozambique between 1 May and 3 June 2014. Children with stunting (HAZ < −2 SD according to the WHO Child Growth Standards in 2006) were considered as cases and those who had a *Z*-score < −2 SD were considered as controls. We collected data related to mothers and children and their environment, and they were assessed in two groups to find a possible association. The software used for data analysis was the SPSS^®^ (version, 21.0) using descriptive statistics, *t*-test, ANOVA, chi-square analyses, bivariate comparisons, and stepwise multiple logistic regression analysis. The results showed that birth weight, mother’s educational status, maternal occupation, living in a rural area, family size, number of children under five years of age in the household, cooking with charcoal, inhabiting wooden or straw housing or housing without proper floors, overall duration of breastfeeding as well as duration of exclusive breastfeeding, and time of initiation of complementary feeding were significantly related to stunting. Thus, appropriate nutritional intervention programmes considering these determinants and the dissemination of knowledge at the population level related to undernutrition are necessary to ameliorate the children´s nutritional status.

## 1. Background

Undernutrition is a major public health problem that increases the global health burden of premature mortality and morbidities during childhood [1,2]. It accounts for 45% of all deaths in children under five years of age. More than two million children under five years of age die each year due to undernutrition around the world [3,4]. 

The prevalence of stunting (chronic undernutrition) (<−2 standard deviation (SD) of low-height-for-age) remains one of the main problems of public health, and a significant proportion of people suffer from moderate or severe undernutrition during early childhood, especially in developing countries, as is the case in Mozambique. The prevalence of stunting is the conventional anthropometric measure that reflects long-term chronic undernutrition, failure of linear growth and multifactorial social deprivation, a long-term response to the prolonged deprivation of food and/or presence of disease. Undernutrition refers to a state resulting from a relative or absolute deficiency of one or more essential nutrients [5,6,7,8,9]. The three main indicators used to define undernutrition, that is to say, stunting, underweight, and wasting, represent different nutritional problems for the child and are measured by the indices of height-for-age, weight-for-height, and weight-for-age, respectively. The Lancet series on maternal and child undernutrition reported critical associations between stunting (length/height-for-age *Z*-score < −2 SD) at age two years and long-term consequences [10,11].

Undernutrition in the first 1000 days post conception represents an important wasting of human potential. In these crucial days, the building blocks are established for the development of the brain and for future growth. Any alteration in this stage has long-term implications, and the damage caused by undernutrition in the early years of life is largely irreversible. In unfavorable environmental conditions, the majority of children will remain locked in their acquired growth channel. The school performance of under-nourished children is reduced. They have lower work capacity and productivity as adults. Later in life they have an increased likelihood of being overweight and developing associated chronic diseases such as cardiovascular disease, diabetes, cancer, and mental health disorders. Although there has been a decrease in prevalence, it has not been accompanied by a commensurate decrease in the number of stunted children due to the increasing population [12,13,14,15,16,17,18]. Over a 10-year period (1990–2010), Africa was the only region where the number of stunted children under five years increased. Projections to 2025 show that the increasing trend is likely to remain [3,8]. ‘The UNICEF’s Annual Reports’ consistently show the prevalence of stunting in Sub-Saharan Africa to be around 30–40%, varying per country or region or urban versus rural environment [17,19,20,21,22]. According to ‘The 2016 Global Nutrition Report’, the prevalence of stunting in Mozambique in children under the age of five years is still high (43.3%) while the underweight (19%) and wasting (5.9%) categories are decreasing [23].

Even though the problem of stunting in Mozambique has been well documented, its specific determinants are not clearly understood. In addition, case-control or cohort studies have not been conducted to identify the risk factors of growth retardation in the Tete province. There is also inconsistency across studies regarding the determinant factors behind stunting in childhood. Therefore, this study attempts to investigate the major socio-economic, demographic, health, and environmental determinants of stunting among infants aged 0–59 months from the central region of Mozambique, i.e., Tete province. The research results obtained would be helpful to healthcare providers in designing and implementing appropriate interventions to improve the growth of children in the central region of Mozambique and other similar regions in the countr, and will enable the government and non-governmental agencies to formulate appropriate policies and initiate intervention strategies for the wellbeing of the population.

## 2. Materials and Methods

### 2.1. Study Area and Population

A case-control study was conducted among children under five years of age between 1 May and 3 June 2014 in the Tete province, Mozambique. The province is located in the central region of Mozambique, bordering Zambia on the North and Zimbabwe on the East, and is the location of the huge Cahora Bassa Dam and the city of Tete. It covers an area of 100,724 km^2^. According to ‘The 2014 Annual National Institute of Statistics’s Report’ [24], the province has a population of 2,500,000 individuals; approximately 20.6% are under the age of five. 

The health system in Mozambique is provided by the Ministry of Health through hospitals, health centres, and health posts. There are three levels of organization of health; the national, provincial, and, finally, the district level. The lowest level of care is provided by health posts. Between the central hospitals and the health posts there are other types of health dispensing units such as health centers, rural hospitals, and provincial or general hospitals [25]. Undernutrition is a very common problem in the region, and the prevalence of stunting is 52% [22]. The study population included children aged 0 to 59 months from two healthcare centres of districts 2 and 3 at the data collection period.

### 2.2. Sample Size Calculation and Sampling Procedure

The sample size was calculated using OpenEpi (Open Source Epidemiologic Statistics for Public Health) version 3.5.4 [26] using formula of two population proportions by assuming the proportion of stunting to be 43.3% based on results from ‘The UNICEF’s Annual Report’ (52% in the Tete province) [22]. In this regard, a 5% level of significance, a power of 80%, and a ratio case to control of 1:2 was assumed. The total sample size was 282 (102 cases and 180 controls). Based on the above assumptions, with an additional 15% added for non-response, the total sample size was 324 children with 110 for cases and 214 for controls. We used a conservative design effect of two to adjust for a complex survey design. The reason for this is that two steps were required to reach or identify the study participants. 

A consecutive sampling technique was used to select the participants until the calculated sample size was attained. All children aged 0 to 59 months visiting healthcare centres 2 and 3 during the data collection period were measured for their height for age *z*-score and categorized as stunted or not stunted. Under-nourished children were first identified and then selected as cases. The cases were stunted children aged 0 to 59 months with height-for age *z*-scores (HAZ) below −2 SD according to the World Health Organization (WHO) Child Growth Standards in 2006 [10]. They were categorized as severely stunted if the HAZ was <−3 SD. The controls were children aged 0 to 59 months without stunting, apparently healthy and living in the community where cases resided. They are considered to be apparently healthy if the mothers/caretakers perceived the child to be healthy and declared no symptoms of disease such as fever, coughing, difficulty of breathing, or diarrhoea and without undernutrition selected from the same healthcare centres. 

Children with mental retardation, physical challenges, serious illnesses, preterm infants (less than 37 weeks of gestation), children suffering from wasting and an exacerbation of chronic undernourishment, and those with mild chronic undernutrition were excluded from the study. After measurements, mothers of the respondents were interviewed by the principal investigator based on a validated questionnaire. Only children accompanied by their own mothers were recruited to avoid recall bias.

### 2.3. Data Collection and Quality Assurance

A two-day training session was given to nine data collectors and one supervisor. The training focused on the questionnaire and the anthropometric measurements. The questionnaire included the demographic characteristics of the children and their caregivers/mothers such as the sex of the child, the age of the child and their mother, the level of education, occupation, and care practices. To minimize recall bias and measurement bias we have offered instructions to the data collector and a supervisor through a two-day training session focused on the survey and anthropometric measurements. 

The initial design of the survey was conducted in Spanish, based on the literature developed for similar purposes and finally translated into Portuguese (the official language) after being adapted to the local context. It also included a physical examination and an anthropometric measurement. Both open and closed questions were used. On average, the interview lasted 30–40 min. The data collection instruments were tested previously and validated. The height and weight of each child were recorded using the standard procedure [27]. 

The height of the subject was recorded with an accuracy of 0.10 cm with the help of a rod out of the anthropometer, with the head held in the Frankfort horizontal plane. Height was measured for children above two years of age, while length for children below two years using a length/height board. The weight of the children was taken with a portable weighing scale with an accuracy close to 0.1 kg, using minimum clothing and with bare feet, and the device was calibrated frequently.

### 2.4. Data Processing and Analysis

The data were analyzed statistically using the Statistical Package for Social Sciences (SPSS^®^ Inc., Chicago, IL, USA; version 21.0). The descriptive statistical analysis of the data obtained is described in terms of mean and standard deviation (SD). The homogeneity of variance was tested using Levene’s test of equal variances. For all anthropometric variables, it was observed that the *p*-value was statistically significant (*p* < 0.05). Normality was tested and also specifies gender using the Shapiro-Wilk test for each one of the anthropometric variables, and it was noted that the *p*-values were not statistically significant (*p* > 0.05). An independent sample *t*-test was performed to assess gender differences in the anthropometric variables. Analysis of variance (ANOVA) using the Scheffé post-hoc test was applied to evaluate the mean differences by age in the anthropometric variables. Chi-square analysis (χ^2^) was used to assess the differences in the age-specific and general prevalence of stunting between the sexes. 

A binary logistic regression analysis was conducted to estimate the odds ratios (OR), and 95% confidence intervals (CIs) were used to evaluate the possible differences and the associated risk factors among children with height-for-age *z*-scores less than −2 SD (HAZ < −2 SD) and normal height-for-age *z*-scores (HAZ > −2 SD). The binary logistic regression analysis allowed the creation of categorical dependent variables, and the odds were obtained by comparing these scors with the reference category in the analysis of the univariate independent model. To create dichotomous dependent variables (stunted growth versus normal), the normal code is coded as (0) and the delay of growth was coded as (1) in the regression models. 

The multiple logistic regression analysis by stages (in advance) in the conditional model was also carried out to determine the most effective predictive variables from the variables considered in the binary logistic regression analysis. A stepwise backward elimination approach was applied, and collinearity was tested in the final model and reported. The odds ratios with 95% CIs were calculated in order to assess the adjusted risk of independent variables, and those with *p* < 0.05 were retained in the final model [28,29].

### 2.5. Ethical Considerations

Ethical approval was provided by the district health authorities and the Ethics Committee of the Tete Regional Hospital, Mozambique. All the participants involved in the study were informed about the nature of the study, the research objectives, and theconfidentiality of the data. The participation of the subjects was completely voluntary in nature, and verbal consent was obtained from each mother before their children were recruited into the study. All the subjects were free from any physical deformities and not suffering from any disease at the time of data collection. To avoid any selection bias, the subjects were examined for any nutritional deficiencies and related disorders. 

Any previous histories related to medical and surgical episodes were also taken into consideration during the time of examination. Persons eligible to participate in the study were not offered a monetary incentive for participation. In the cases where the respondent was illiterate, we asked a literate person from the community to read out the consent form and explain it to the head of the family. Then we obtained the thumb print of the respondent. In those cases, the person who read the consent form also signed as a witness. 

The research procedures were consistent with the Declaration of Helsinki [30]. Interviews were administered after obtaining informed consent. The protocol was reviewed by a small group of experts who had experience working in centers of recovery and nutritional education and was amended based on their recommendations.

## 3. Results

### 3.1. Sociodemographic Characteristics

The size of the sample was 282 patients (*n* = 282) from 13 districts. Of these, 69.2% (*n* = 195) lived in an urban area and 22.7% (*n* = 64) in a rural area. Regarding distribution by sex, 57.4% of the sample and of the cases were boys, with an average age of 42 months ± SD of 18.3 (range: 0–59). Differences observed between percentages of stunting and normally nourished children according to sex were statistically significant (*p* < 0.05). As to the children’s age, it was higher in the group of stunting. The mean ages ± SD for the cases and controls was 43.3 ± 18.5 and 40.9 ± 18.2, respectively (*p* = 0.31). When they were grouped by age intervals, a higher percentage of stunting was shown in those under six and those over 24, but the results were not significant either (*p* > 0.05). 

The mean pregnancies ± SD for the cases and controls were 3.6 ± 1.8 and 1.6 ± 0.9, respectively, while their mean birth weights ± SD were 2.8 ± 0.4 and 3.1 ± 0.3 kg, respectively (*p* < 0.001). The findings highlighted that there were significant associations between undesirable growth of the subjects and their mothers’ educational level (*p* < 0.001). The mean age of the mothers in their first pregnancy ± SD for the cases and controls was 16.8 ± 1.8 and 20.5 ± 2.5, respectively (*p* < 0.05) (Table 1).

### 3.2. Feeding Habits and Practices and Findings in the Examination of Malnourished Children

Both the duration of breastfeeding (17.4 ± 5.4 and 11.4 ± 6.1 months) and the introduction of beikost (7.6 ± 2.3 and 5.3 ± 2.3 months) were higher in the group of normally nourished against the group of malnourished, being significant in both cases (*p* < 0.001). The early incorporation of these foods was associated with a higher risk of suffering stunting, and the observed differences were statistically significant, except for eggs and legumes. 

Moreover, the frequency of intake of foods was also analysed. No relevant differences were observed between groups in regards to the consumption of foods such as yoghurt, pasteurized milk, vegetables, potatoes, chicken, and fish. Only a small percentage had yoghurt regularly and pasteurized milk with sugar occasionally. The majority ate vegetables, potatoes, chicken, and fish every day on a regular basis. However, there were statistically significant differences observed regarding the intake of other foods. The results obtained from the comparison of housewives withthose who work outside the home, showed that the condition of having a job outside the home increases the risk of a child suffering undernutrition (*p* < 0.05). The most frequent findings in physical examination of malnourished children were changes in the skin (60.8%), loss of muscle mass (57.8%), alterations of the oral mucosa (57.8%), alterations of the subcutaneous cellular tissue (56.9%), alterations in hair and nails (48%), ocular alterations (47.1%), distended abdomen (30.4%), and neurological disorders (16.7%) (Table 1).

### 3.3. Characteristics of the Environment

Other relatives lived at home with the malnourished in 65.7% of the cases, whilst only in 9.4% of the control group had relatives living with them (*p* < 0.001) (Table 1).

### 3.4. Simple and Multiple Logistic Regression Analyses

The results of the univariate binary logistic regression analysis and the associations of sociodemographic variables on the prevalence of stunting among children are shown in Table 2. The following factors remained and were incorporated into the regression model; the child´s age in months, birth weight, mother’s educational status, maternal occupation, living in a rural area, family size, number of children under five years of age in the household, cooking with charcoal, and inhabiting wooden or straw housing or housing without proper floors (Table 2).

Finally, the factors associated with stunting were examined in a multiple logistic regression model. A stepwise backward trend was retained in the final model. The odds ratios with 95% CIs were calculated in order to assess the adjusted risk of independent variables, and those with *p* < 0.05 were retained in the final model. It was observed that the child’s sex, birth weight, living in a rural area, having siblings under five years of age, living in houses made of straw and wood with soil floor, and homes where other relatives lived too, maintain statistically significant differences (*p* < 0.05).

Sociodemographic factors were significantly associated with chronic undernutrition in children. The relationship between gender and chronic undernutrition was statistically significant [Adjusted Odds Ratio (AOR) = 4.57, 95% CI = (2.06–10.12), *p* < 0.05]. Birth weight was significantly associated with stunting [AOR = 19.99, 95% CI = (5.8–68.85), *p* < 0.001] and reported a lower risk of stunting. Children living in urban areas significantly reported a lower risk of stunting than those in rural areas [AOR = 138.0, 95% CI = (32.38–587.80), *p* < 0.001].

Children living in households with other family members [AOR = 17.3, 95% CI = (7.62–39.12), *p* = 0.001] and living in households with other children less than five years of age were more likely to develop stunting [AOR = 28.42, 95% CI = (11.93–67.70), *p* < 0.001]. Similarly, stunting was also related to living in households which used charcoal for cooking, households made of straw and wood [AOR = 3.10, 95% CI = (1.53–6.26), *p* = 0.002], or those households which did not have a proper floor [AOR = 17.26, 95% CI = (5.87–50.75), *p* = 0.001] (Table 3).

## 4. Discussion

Linear growth is the best overall indicator of children’s well-being and provides an accurate marker of inequalities in human development. This is tragically reflected in the millions of children throughout the world who do not achieve their full potential of linear growth due to suboptimal health conditions and inadequate nutrition and childcare [31]. The nutrition assessment of the vulnerable segments of the population should be emphasised, not only for the identification of nutritional risks but also for the improvement of existing health situations. Currently, the nutritional scenario of the developing countries has been changing radically over the past two decades, experiencing the double burden of malnutrition (both under and overnutrition) due to changes in socio-economic and demographic transition, dietary habits, lifestyle modification, and increasing risks of non-communicable diseases [32,33].

This study assessed the child growth situation in the central region of Mozambique (Tete province) and identified sociodemographic, health, and environmental variables as important determinants of stunting of children under five in the Tete province. The findings showed that the overall prevalence of stunting was observed to be 36.2% using the proposed WHO reference (WHO 2006). The rate of 52% stunting in the Tete province is much higher than the 40% critical public health thresholds. This estimation is almost similar to the 43% level reported by the 2007 Demographic Health Survey (DHS) for this age group, suggesting that not much has changed between 2007 and 2016 [34].

The study findings depicted the sex of child to be a strong determinant of childhood stunting. Previous studies from Sub-Saharan Africa have reported mixed findings on the effects of sex, with some suggesting that males are more affected by undernutrition compared to females, while others reported otherwise [35,36,37,38]. In this study, males were more likely to be stunted. This is in line with the findings of a meta-analysis of DHS of 16 Sub-Saharan African countries [39], as well as the 2011 Mozambique DHS [40] and the 2010 DHS in Eastern Africa [41]. Studies in Sub-Sahara African countries have reported girls to be more recognized, first because of their high value in agriculture and secondly due to the fact that they are seen as an investment, especially among the low socio-economic class, thus leading to more care and dietary preferential treatment [39]. Considering that over 80% of Mozambique is agriculturally based and that the majority of the population is socio-economically constrained, i.e. an estimated 61% are below the poverty line of $1.25 per day [42], this notion may explain why males are more malnourished. Additionally, epidemiological evidence depicts boys to be biologically more vulnerable to morbidity [43,44], and, in a setting like Mozambique where morbidity incidences are high, this probably exerts considerable effects on boys.

Childhood stunting was found to progressively rise with an increase in age up to the age of 24 months. A similar trend has been reported in a number of previous studies in developing countries [18,37,45,46,47,48]. The decreasing immune protective effects of breast milk coupled with increasing exposure to contaminated complementary foods, culminating in the onset of infectious diseases along with increasing nutrient requirements, explains the trend [49,50,51,52]. In line with a number of studies from settings like Central-Eastern Africa (Kenya) [36], Turkey [53], and Eastern Africa (Burundi) [41] along with the recent lancet series, which put children born with low birth weight at a 20% risk of stunting [3], low birth weight children in this study were at a higher risk of stunting. Research shows that low birth weight babies are born with low reserves of vital growth nutrients; vitamin A, zinc, and iron [54]. Therefore, they depend on breast milk to cover these deficiencies.

However, as the amount of these nutrients present in breast milk mainly depends on maternal intake and nutritional status, low birth weight children are at a risk of not meeting the recommended amounts if maternal intake is inadequate. Additionally, smaller than average babies experience feeding problems, a factor involved in undernutrition, even in this study [55]. A number of studies have shown maternal education to provide protective effects against all under-nutrition indicators in children [36,45,46,56,57,58,59,60]. The findings of this study are inconsistent with the results of the studies in terms of growth retardation and underweight children, with which no association was found. Particularly, the results disagree with the 10 years schooling threshold reported in a recent study conducted across the three countries of Malawi, Zimbabwe, and Tanzania [61], as protective effects were only found for secondary/tertiary education with no effects for primary completion. As reported in other settings [59,62], 10 years of schooling may represent an opportunity of achieving a higher paying job, meaning better access to the market and leading to better feeding practices. Additionally, these mothers may have better child and healthcare knowledge, more health seeking behaviours, lower fertility rates, and access to better medical care. Certainly, in this study, a significant proportion of children whose mothers had secondary/tertiary education followed a diet that met the recommended dietary diversity, in comparison with their counterparts whose mothers were uneducated. 

The study findings depict no association between maternal occupation and any of the undernutrition indicators. This is contrary to a recent systematic review on determinants of undernutrition in Sub-Saharan Africa [38], as well as other studies from Malaysia [63] and Ethiopia [64], but in agreement with findings of Adekanmbi and Kayode [45] from Nigeria. As the majority of mothers are in the agricultural sector, which is largely on the subsistence level, it could be that there contribution towards the household income is too low to influence household decisions like feeding practices.

Children of undernourished mothers are more likely to be stunted. Similar findings have been documented in Tanzania [56] and other low-income countries such as Nigeria [45] and Ethiopia [18,58]. Though a group of evidence shows that maternal nutritional status has no effect on the composition of breast milk, this may not be the case for some key growth micronutrients like vitamin A, iodine, riboflavin, thiamine, and others [7]. Consequently, maternal deficiency means infant deficiency, especially for vitamin A, the stores of which are low at birth. Additionally, it is suggested that it is a risk factor for foetal growth restriction, culminating in low birth weight [54,65]. Low birth weight means more depletion of already low stores of key growth nutrients, which the mother’s breast milk continues to lack due to poor maternal nutritional status, thus resulting in a prolonged lack in these children. The findings of this study support those of previous ones that have consistently shown household wealth status to have a substantial protective effect against all the indicators of child undernutrition [38,45,57,58,66]. In countries where contrary findings have been reported, scaling up access to quality public health services was the point that marked the difference [67].

Therefore, in settings where there is still a discriminative access to quality health services, improved sanitation facilities, and water sources, this all depends on wealth status, showing that the latter still remains a major determinant of health and nutritional status. This is likely to be the case in Mozambique, hence explaining the increasing trend of protective effects against child under-nutrition as household wealth status increases. Additionally, a higher wealth status guarantees better access to adequate nutritious food supplies. The odds of stunting were higher among children from households with more than one child under five years of age. This could be attributed to inadequate feeding practices; both breast and complementary, i.e. mothers may be unable to optimally meet the breastfeeding recommendations, especially when these children are single births. They may stop breastfeeding upon reconceiving.

This also comes with a higher risk of low birth weight, not only because a mother conceives before fully recovering from the nutrition burden of the last pregnancy but also due to the fact that the lactation burden is also comparatively high. According to Dewey [68], the nutritional burden of lactation etimated at an increase of 25% energy needs, 54% protein, and 0–93% micronutrient depending on the micronutrient in question, is even greater than that of pregnancy (13% energy needs, 54% protein, and 0–50% micronutrients). Therefore, a pregnancy that overlaps with lactation owing to a short birth interval is associated with a considerable depletion of maternal reserves, negatively affecting both the mother and the growing foetus [69]. As a result, it is not a coincidence that the recent Lancet series associated short birth intervals with a low birth weight risk of 1.65 [3]. A study by Adekanmbi and Kayode [45] found increased odds of stunting among children with birth intervals below 24 months. The increased risk is not only among single births but also among multiple births.

A household larger than the average size in this setting (five members) was associated with higher odds of childhood stunting. This is in line with a number of studies that have found a significant relationship between bigger household sizes and childhood undernutrition [57,66,70]. A large household size suggests increased competition for scarce resources [71]. This becomes a major constraint in a low socio-economic settings like Mozambique. Household hygienic practices such as access to safe water, hand washing using soap, and other sanitation practices attenuate diarrhoea and other morbidity related risks, which have a considerable effect on child growth [72,73]. Therefore, improvements in hand washing practices and water quality are considered to be vital steps to prevent environmental enteropathy and thereby reduce the risk of undernutrition [74].

In response to reducing child undernutrition and other complex challenges, and in the context of the United Nations Development Assistance Framework (UNDAF) 2017–2020, The Government’s five-year Plan 2015–2019, the Sustainable Development Goals (SDGs), and the UNICEF Strategic Plan, UNICEF and partners in consultation with the Mozambique government have developed a four-year programme to work for change for Mozambican children and women. With a strategic mix of interventions, the programme will drive progress towards the realization of children’s rights by: (a) ensuring that children have access to critical social services and supplies to meet their basic needs; (b) promoting behavioural and social change; (c) advocating for changes in policies affecting children and the corresponding allocation and use of domestic resources; and (d) advancing systemic changes in health, education, water and sanitation, and protection to address children’s vulnerability to poverty and reduce persistent disparities. Programmatic focus is on the whole child, particularly the poorest and most marginalized. In Mozambique, however, this means many children because vulnerability is deep and multi-dimensional poverty affects all the bottom four wealth quintiles, both in urban and rural contexts.

The widespread presence of UNICEF on the ground and its knowledge and access to the government make it well positioned to make valuable contributions to the children’s agenda in Mozambique. After many years focusing on access to services, it is clear that quality and equity are fundamental missing links in service delivery, along with closing gaps in access, especially for the most vulnerable and marginalized populations. Across programme areas, renewed efforts are needed to gather data and evidence for child-centred programming and to advocate for the use of evidence in policy and budget decision making. UNICEF will continue to catalyse partnerships, bringing together the government, academia, civil society, the private sector, and citizens, including children and young people, for common action in support of child-centred, gender-focused, and equitable results.

Nutrition is a flagship issue for the United Nations and UNICEF in Mozambique, and a multi-sectoral approach is urgently needed at national and sub-national levels. The programme will work to develop replicable, sustainable, integrated models, tied to government systems and capacities, with clearly defined roles for long-term action. UNICEF will support the government and partners to improve nutritional status among children in the first 1000 days by focusing interventions on adolescent girls and lactating and pregnant women in order to reduce stunting, especially in high burden provinces.

In addition, UNICEF will leverage partners to ensure optimal nutrition among school-aged children. Interventions will concentrate on fostering multi-sectoral partnerships, leveraging increased resource allocation, developing legislation, strategies and plans, supporting the implementation of nutrition policies, strengthening sub-national coordination, and supporting behavioural change at the community level to improve infant and young child nutrition. Success will rely on multi-sectoral action, working closely with United Nations sister organizations, the Technical Secretariat for Food Security and Nutrition, the Nutrition Partners Forum, the Ministry of Health, and civil society. The country programme 2017–2020 and the UNDAF include a robust monitoring framework, focused on the continual measurement of progress against key benchmarks. Additionally, the UNICEF programme falls within the Eastern and Southern Africa Regional Office regional compact, allowing for the measurement of commonly agreed regional indicators to support corporate priority results. Support for improved programme and humanitarian performance monitoring and capacity strengthening for UNICEF and partners will be central to the implementation and monitoring strategy.

UNICEF will continue its work with partner ministries to ensure that relevant and reliable data are regularly produced at the national and sub-national levels and will ensure the ongoing measurement of the situation of children. Key national data milestones in the forthcoming years will include the comprehensive violence against children study (2016), the national census (2017), and a demographic and health survey (2018–2019). 

At the same time, UNICEF will support evidence-based planning and budgeting at national and decentralized levels, aiding the government and its partners to identify excluded children and orient budgets and plans to support their development. Special attention will be given to monitoring the impact of natural disasters and the political and economic crises on the situation of children. Major programme evaluations will include evaluation of the pivotal child health workers programme, the community-led total sanitation approach, and the soon-to-be-implemented child grant. Finally, in coordination with the government and its partners, UNICEF will hold annual reviews and mid and end-term evaluation exercises to ensure that the programme design remains optimally focused to achieve an impact on behalf of the children [75].

The data used here may have a number of limitations on the outcome of this study. Although there are many factors associated with stunting, as indicated by various studies in different countries, this analysis is only undertaken to explore a few of the sociodemographic, health, and environmental factors in Mozambique. Also, this study does not make a comparative evaluation of growth retardation between rural and urban households in the country, and there is not a periodic publication of reports and approved resources. However, taking the limitations into consideration, the study findings are credible in a more specific way as they are also consistent with the existing literature in this field. Through this research, an analysis of children’s nutritional status in relation to demographic, socio-economic, and feeding practices is presented. It is hoped that the finding of this study will identify the risk factors for stunting among children under the age of five years in the Tete province.

The situation of stunting in Mozambique (43.3%) is above 40% in all provinces, but the situation is especially severe in the Tete province (52%). Study findings reaffirmed that child growth is multidimensional and identified socio-economic, demographic, health, and environmental variables as important determinants of stunting for children under five in the Tete province. Similar to previous studies, undernutrition in children increased with age and was elevated in children with low birth weight and in males. The findings from this study have some relevant policy implications for the child health programme in Mozambique. There is a clear need for intervention to reduce economic inequalities and ultimately poverty among the populace. Programs with a special focus on child health and nutrition should be organized, particularly for women in communities with high illiteracy rates, as a short-term solutions aimed at increasing low literacy levels in the Tete province. Any intervention by governmental and non-governmental organizations that target improving the nutritional status of children under five should consider regions with a high rate of childhood stunting so as to avoid under-coverage of the regions that deserve it.

## 5. Conclusions

To effectively respond to the myriad of dietary and undernutrition challenges that lie ahead, a coordinated multi-sectoral enabling environment and response is necessary. This requires dialogue between all relevant sectors and actors, including policymakers, development actors, civil society, donors, the private sector, consumers, and producers. There are many avenues to achieve this dialogue across different platforms. However, we must institute better governance and accountability.

## Figures and Tables

**Table 1:** Distribution of stunting status by characteristics of mothers and children and their environment (*N* = 282). Data is expressed in frequencies (%) or average ± SD (standar deviation).

	Chronically Malnourished	Normally Nourished	Total	*p*-Value
	*N* = 102	*N* = 180	*N* = 282	
	*n* (%)	*n* (%)	*n* (%)	
	$\bar{X}$ ± σ	$\bar{X}$ ± σ	$\bar{X}$ ± σ	
***Sex***				
Male	79 (77.5)	83 (46.1)	162 (57.4)	0.001 ***
Female	23 (22.5)	97 (53.9)	120 (42.6)	
***Age (months)***	43.3 ± 18.5	40.9 ± 18.2	-	0.31
***Area of origin***				
Urban	20 (19.6)	175 (97.2)	195 (69.1)	0.001 ***
Periurban	20 (19.6)	3 (1.7)	23 (8.2)	
Rural	62 (60.8)	2 (1.1)	64 (22.7)	
***Reason for consultation***				
Respiratory infection	17 (16.7)	10 (5.6)	27 (9.6)	0.001 ***
Gastrointestinal infection	43 (42.2)	90 (50.0)	133 (47.2)	
Fever	33 (32.4)	78 (43.3)	111 (39.4)	
Weight control and vaccination	9 (8.8)	2 (1.1)	11 (3.9)	
***Routine checkups ^a^***	16 (15.7)	157 (82.7)	173 (61.3)	0.001 ***
***Completed vaccination schedule ^b^***	56 (54.9)	179 (99.4)	235 (83.3)	0.001 ***
***Age of beikost ^c^***				
<6 months	75 (73.5)	54 (30)	129 (45.7)	0.001 ***
≥6 months	27 (26.5)	126 (70)	153 (54.3)	
***Time in months***				
Cereals ^d^	5.51 ± 2.42	7.76 ± 2	-	0.001 ***
Fruit	7.01 ± 3.02	7.92 ± 2.04	-	0.003 **
Vegetables	7.5 ± 3.31	8.28 ± 2.1	-	0.02 *
Cow’s milk	0.93 ± 4.22	1.04 ± 4.56	-	0.85
Cow’s meat (beef)	13.3 ± 6.74	11.48 ± 9.33	-	0.06
Fish	11.85 ± 5.04	13.93 ± 5.51	-	0.002 **
Chicken	13.4 ± 5.75	15.32 ± 5.34	-	0.01 *
Eggs	9.97 ± 5.37	10.39 ± 4.43	-	0.48
Yoghurt	0.9 ± 3.24	1.06 ± 2.76	-	0.66
Legumes	7.77 ± 4.06	8.18 ± 2.37	-	0.35
Juices	3.83 ± 5.51	6.99 ± 3.43	-	0.001 ***
***Siblings < 5 years old***	78 (76.5)	13 (7.2)	91 (32.3)	0.001 ***
***Type of home***				
Straw and wood	45 (44.1)	35 (19.4)	80 (28.4)	0.001 ***
Clay bricks	53 (52)	79 (43.9)	132 (46.8)	
Others	4 (3.9)	66 (36.7)	70 (30.6)	
***Type of floor***				
Soil	98 (96.1)	63 (35)	161 (57.1)	0.001 ***
Concrete	4 (3.9)	117 (65)	121 (42.9)	
***Access to drinking water***	-	52 (28.9)	52 (18.4)	0.001 ***
Cooking fuel *				
Coal	19 (18.6)	128 (71.1)	147 (52.1)	0.001 ***
Wood	83 (81.4)	30 (16.7)	113 (40.1)	
Gas	-	22 (12.2)	22 (7.8)	

* *p* < 0.05 (Chi-square), ** *p* < 0.01, *** *p* < 0.001; ^a^ Those who have visited the healthcare centre at least once in the last year have been considered; ^b^ Completed vaccination schedule for the child’s age according to the Ministry of Health in Mozambique; ^c^ Children aged six months or over were considered; ^d^ Cereals consumed in form of ‘xhima’ or ‘papinha’.

**Table 2:** Binary logistic regression (BLR) analysis and associations of sociodemographic variables with stunting.

	Binary Logistic Regression Analysis Univariate Model ^†^				*p*-Value
	Cases	Controls	Crude OR	95% IC	
	*N* = 102	*N* = 180			
	*n* (%)	*n* (%)			
***Sex***					
Male	79 (77.5)	83 (46.1)	4.01	(2.32; 6.95)	0.001 ***
Female (ref.)	23 (22.5)	97 (53.9)	-		
***Area of origin***					
Urban	40 (39.2)	178 (98.9)	137.95	(32.38; 587.65)	0.001 ***
Rural (ref.)	62 (60.8)	2 (1.1)	-		-
***Birth weight***					
≤2.50 kg	36 (35.3)	4 (2.2)	23.86	(8.18; 69.65)	0.001 ***
>2.50 kg (ref.)	66 (64.7)	175 (97.8)	-		-
***Mothers’ education***					
No education/primary	102 (100)	123 (68.3)	-		-
≥Secondary (ref.)	-	57 (31.7)	-		-
***Mother occupation***					
Housewife (ref.)	76 (74.5)	167 (92.8)	-		-
Others	26 (25.5)	13 (7.2)	0.23	(0.11; 0.47)	0.304
***Extended family household***					
Yes	67 (65.7)	17 (9.4)	18.36	(9.63; 35)	0.001 ***
No (ref.)	35 (34.3)	163 (90.6)	-		
***Siblings under 5 years old***					
Yes	78 (76.5)	13 (7.2)	41.75	(20.19; 86.33)	0.001 ***
No (ref.)	24 (23.5)	167 (92.8)	-		-
***Type of home***					
Straw and wood	45 (44.1)	35 (19.4)	3.27	(1.91; 5.60)	0.002 ***
**Others (ref.)**	57 (55.9)	145 (80.6)			
***Type of floor***					
Soil	98 (96.1)	63 (35.0)	45.5	(15.99; 129.46)	0.001 ***
Concrete (ref.)	4 (3.9)	117 (65.0)	-		-
***Access to drinking water***					
Yes (ref.)	-	52 (28.9)	0.56	(0.50; 0.625)	-
No	102 (100)	128 (71.1)	-		
***Cooking fuel***					
Coal (ref.)	19 (18.6)	150 (83.3)	-		-
Wood	83 (81.4)	30 (16.7)	0.05	(0.024; 0.086)	0.001 ***

*** *p* < 0.001; ref.: reference category; OR—odds ratio; CI—confidence interval; ^†^—binary logistic regression analysis considering effect of one explanatory/predictor variable.

**Table 3:** Unadjusted and adjusted odds ratios (OR) (95% CI) for stunted children aged 0–59 months.

	Cases	Controls	Crude Odds Ratio OR	Adjusted Odds Ratio ^a^ aOR	*p*-Value
	*N* = 102	*N* = 180			
	*n* (%)	*n* (%)	95% CI	95% CI	
***Sex***					
Male	79 (77.5)	83 (46.1)	4.01 (2.32; 6.95)	4.57 (2.06; 10.12)	0.001 ***
Female (ref.)	23 (22.5)	97 (53.9)	-	-	
***Area of origin***					
Urban	40 (39.2)	178 (98.9)	137.95(32.38; 587.65)	138 (32.38; 587.80)	
Rural (ref.)	62 (60.8)	2 (1.1)	-	-	0.001 ***
***Birth weight***					
≤2.50 kg	36 (35.3)	4 (2.2)	23.86 (8.18; 69.65)	19.99 (5.80; 68.85)	0.001 ***
>2.50 kg (ref.)	66 (64.7)	175 (97.8)	-	-	
***Mothers’ education***					
No education/1 ^a^	102 (100)	123 (68.3)	-	-	-
≥Secondary (ref.)	-	57 (31.7)	-	-	
***Mother occupation***					
Housewife (ref.)	76 (74.5)	167 (92.8)	-	-	
Others	26 (25.5)	13 (7.2)	0.23 (0.11; 0.47)	2.37 (0.46; 12.21)	0.304
***Extended family household***					
Yes	67 (65.7)	17 (9.4)	18.36 (9.63; 35)	17.27 (7.62; 39.12)	0.001 ***
No (ref.)	35 (34.3)	163 (90.6)	-		
***Siblings under 5 years old***					
Yes	78 (76.5)	13 (7.2)	41.75 (20.19; 86.33)	28.42 (11.93; 67.70)	0.001 ***
No (ref.)	24 (23.5)	167 (92.8)	-	-	
***Type of home***					
Straw and wood	45 (44.1)	35 (19.4)	3.27 (1.91; 5.60)	3.10 (1.53; 6.26)	0.002 **
**Others (ref.)**	57 (55.9)	145 (80.6)			
***Type of floor***					
Soil	98 (96.1)	63 (35.0)	45.5 (15.99; 129.46)	17.26 (5.87; 50.75)	0.001 ***
Concrete (ref.)	4 (3.9)	117 (65.0)	-	-	
***Access to drinking water***					
Yes (ref.)	-	52 (28.9)	0.56 (0.50; 0.625)	-	
No	102 (100)	128 (71.1)	-		
***Cooking fuel***					
Coal (ref.)	19 (18.6)	150 (83.3)	-	-	
Wood	83 (81.4)	30 (16.7)	0.05 (0.024; 0.086)	0.055 (0.024; 0.124)	0.001 ***

** *p* < 0.01, *** *p* < 0.001; CI—confidence interval; significance considered when *p* < 0.05 and if the OR and adjusted odds ratios (aOR) estimate did not cross the null for 95% CI. ^a^ OR adjusted for child‘s sex and area of origin.

## Abbreviations

SD	Standard deviation
OR	Odds ratio
aOR	Adjusted odds ratio
CI	Confidence interval
UNICEF	The United Nations Children’s Fund
UNDAF	United Nations Development Assistance Framework
WHO	World Health Organization
FAO	The Food and Agriculture Organization
HAZ	Height for age *z*-score
DHS	Demographic Health Survey
SDGs	Sustainable Development Goals
